# Metabolic stress exacerbates Ca^2+^ dysregulation and arrhythmias in catecholaminergic polymorphic ventricular tachycardia

**DOI:** 10.1113/EP094053

**Published:** 2026-10-11

**Authors:** Valeria Gomez, Arpita Deb, Zhuoxin Li, Yujia Cheng, Benjamin K. Fischer, Jinying Yang, Jeongmin Lee, Bjorn C. Knollmann, Zhiyong Cheng, Bin Liu

**Affiliations:** ^1^ Food Science and Human Nutrition Department University of Florida Gainesville Florida USA; ^2^ Department of Biological Sciences Mississippi State University Starkville Mississippi USA; ^3^ Department of Applied Physiology and Kinesiology University of Florida Gainesville Florida USA; ^4^ Department of Medicine Vanderbilt University School of Medicine Nashville Tennessee USA

**Keywords:** arrhythmias, calcium signalling, diabetes

## Abstract

Catecholaminergic polymorphic ventricular tachycardia (CPVT) is a genetic arrhythmic syndrome caused by mutations in the sarcoplasmic reticulum (SR) calcium (Ca^2+^) release channel RyR2 or its accessory proteins, including calsequestrin‐2 (CASQ2). RyR2 dysfunction or leak is the hallmark feature of CPVT and directly triggers arrhythmias. Despite this electrical instability, CPVT hearts are typically anatomically normal and lack pathological remodelling. Therefore, we hypothesized that introducing additional stressors, such as metabolic stress, would worsen cardiac arrhythmias and potentially trigger pathological remodelling. To test this hypothesis, metabolic stress was introduced by crossbreeding a CPVT model of CASQ2^−/−^ mice with the Akita mouse, a genetic model of diabetes that also lacks cardiac remodelling. We found that dysregulation of myocyte Ca^2+^ handling was exacerbated in Akita‐CASQ2^−/−^ cells, which exhibited slower Ca^2+^ transient decay kinetics and increased arrhythmogenic Ca^2+^ waves. Akita‐CASQ2^−/−^ mice also displayed more severe arrhythmias, diastolic dysfunction, and cardiac hypertrophy, but without evidence of fibrosis, cell death, or worsened metabolic dysfunction. Finally, mitochondrial reactive oxygen species production was increased in Akita‐CASQ2^−/−^ cardiomyocytes, which may contribute to the more severe pathological phenotype. Overall, our results suggest that metabolic stress can aggravate dysregulated myocyte Ca^2+^ handling and promote cardiac dysfunction in CPVT hearts.

## Abstract (highlights)

- **What is the central question of this study?**
  Does metabolic stress introduced by hyperglycaemia exacerbate cardiac dysfunctions in a Ca^2+^‐dependent genetic arrhythmic syndrome (CPVT), which typically does not exhibit cardiac pathological remodelling?
- **What is the main finding and its importance?**
  Type 1 diabetes‐associated hyperglycaemia significantly exacerbated myocyte Ca^2+^ handling abnormalities and cardiac arrhythmias in a CPVT model. In addition, metabolic stress induced cardiac hypertrophy and contractile dysfunction in the CPVT heart. These findings highlight the critical role of metabolic homeostasis and demonstrate an important interplay between hyperglycaemia and Ca^2+^‐dependent cardiac dysfunction.

## 1 INTRODUCTION

Calcium (Ca^2+^) regulates cardiac contraction and heart rhythm (Bers, 2001). Ryanodine receptor 2 (RyR2), together with multiple accessory proteins, forms a Ca^2+^ release channel complex in the sarcoplasmic reticulum (SR) membrane that controls intracellular Ca^2+^ release and thereby regulates cardiac function (Gyorke & Terentyev, 2008). Under physiological conditions, the RyR2 channel complex remains refractory following systolic Ca^2+^ release. However, pathological modifications can cause the channel complex to recover prematurely from this refractory state, leading to aberrant diastolic Ca^2+^ release (Gyorke et al., 2017). This hyperactivity of the RyR2 channel is commonly referred to as RyR2 leak.

RyR2 leak has been implicated in a spectrum of cardiac dysfunctions, including arrhythmias, heart failure, and metabolic heart disease (Belevych et al., 2013; Deb et al., 2026; Tow et al., 2022). A direct consequence of RyR2 leak is the development of cardiac arrhythmias. Catecholaminergic polymorphic ventricular tachycardia (CPVT) is a genetic arrhythmic syndrome caused by mutations in RyR2 or its accessory proteins, including calsequestrin 2 (CASQ2), calmodulin, and triadin. Most CPVT‐associated mutations render the channel hyperactive or leaky, resulting in spontaneous diastolic Ca^2+^ release. When sufficiently large, these Ca^2+^ release events generate electrogenic currents that trigger ectopic beats (Gyorke, 2009).

CPVT is a life‐threatening arrhythmic syndrome; however, CPVT hearts are typically anatomically normal and do not exhibit the pathological remodelling frequently observed in other cardiac conditions, such as heart failure or metabolic heart disease. It has been hypothesized that the magnitude of RyR2 leak must reach a certain threshold to induce pathological cardiac remodelling. Genetic evidence supports this idea, as increasing SR Ca^2+^ load to promote RyR2 leak results in severe Ca^2+^‐dependent cardiomyopathy (Kalyanasundaram et al., 2013; Liu et al., 2014). In addition, our recent study identified mitochondria as key organelles that translate RyR2 leak into diverse disease phenotypes (Tow et al., 2022). Ablation of the mitochondrial Ca^2+^ uniporter (MCU), a protein responsible for mitochondrial Ca^2+^ uptake, leads to premature death and severe cardiomyopathy in a CPVT model with CASQ2 knockout (CASQ2^−/−^) (Tow et al., 2022). These findings suggest that when combined with additional stressors that exacerbate RyR2 leak, pathological cardiac remodelling may develop in the setting of CPVT.

In addition to genetic mutations, excessive post‐translational modifications of RyR2 can cause acquired channel defects and promote RyR2 leak, thereby contributing to multiple cardiac conditions, including diabetic cardiomyopathy (DbCM) (Shao et al., 2009; Yaras et al., 2005). DbCM is characterized by metabolic stress and altered Ca^2+^ homeostasis. The genetic type 1 diabetes model, the Akita mouse, exhibits a relatively mild cardiac phenotype. Although diabetic, Akita mice do not develop the systolic dysfunction, hypertrophy, or fibrosis observed in some other DbCM models (Basu et al., 2009). This relatively mild phenotype makes the Akita mouse an ideal model to test whether the combination of metabolic stress and RyR2 leak can induce more severe cardiac dysfunction.

Therefore, we crossbred the Akita model with the CPVT model of CASQ2 knockout to introduce metabolic stress into a classic model characterized by RyR2 leak. We hypothesized that Ca^2+^ handling defects and cardiac arrhythmias would be exacerbated in this combined model. Additionally, we investigated whether the combination of metabolic stress and RyR2 leak can induce more severe cardiac pathophysiology.

## 2 METHODS

### 2.1 Ethical approval

All experiments and animal procedures were conducted in accordance with ethical guidelines approved by the Institutional Animal Care and Use Committees (IACUCs) of the University of Florida (approval reference number: IACUC202300000432) and Mississippi State University (approval reference number: IACUC‐20‐337), and were in compliance with the animal experimentation policies of *Experimental Physiology*. The study was conducted in accordance with the *Guide for the Care and Use of Laboratory Animals* established by the U.S. National Institutes of Health (NIH Publication No. 85‐23, revised 2011). Mice were housed in an AAALAC‐accredited animal facility with ad libitum access to food and water and were cared for by university animal care staff. Only non‐survival surgical procedures were performed in this study. Mice were anaesthetized with 3% isoflurane in oxygen delivered via a precision vaporizer. Following thoracotomy and heart excision, mice were euthanized by exsanguination.

### 2.2 Animal model

Age‐ and litter‐controlled male mice (3 months old) were used in all experiments. The Akita model was obtained from The Jackson Laboratory (Bar Harbor, ME, USA; Stock No. 003548) and crossbred with the CASQ2^−/−^ model to generate Akita‐CASQ2^−/−^ mice. Mouse genotypes were verified by polymerase chain reaction (PCR) analysis of tail DNA. The number of mice included in the study was as follows: WT, *N* = 30; Akita, *N* = 31; CASQ2^−/−^, *N* = 28; and Akita‐CASQ2^−/−^, *N* = 32. Only male mice were included because they are more susceptible to hyperglycaemia, diabetes, and related chronic complications, including cardiac dysfunction (Ceylan‐Isik et al., 2006; Le May et al., 2006; Yan et al., 2022).

### 2.3 Echocardiography

In vivo cardiac function was assessed by transthoracic echocardiography using a LOGIQ e NextGen system (Sound Technologies, Carlsbad, CA, USA). Adult mice were lightly anaesthetized with isoflurane during image acquisition. M‐mode images of the left ventricle (LV) were obtained from the parasternal short‐axis view at the level of the papillary muscles. Pulse‐wave Doppler recordings were acquired at the level of the mitral valve leaflet tips. The myocardial performance index (MPI) was calculated as: MPI = (IVRT + IVCT)/ET, where IVRT represents isovolumic relaxation time, IVCT is isovolumic contraction time and ET is ejection time. Measurements were performed as previously described (Min et al., 2015). Image analysis was conducted using ImageJ software (NIH), and values were averaged over 8–10 consecutive cardiac cycles for each animal.

### 2.4 Histological staining

Animal hearts were fixed in 10% phosphate‐buffered formaldehyde (pH 7.4), paraffin‐embedded, and sectioned longitudinally at approximately 5 µm thickness. As previously described, cardiac tissue sections were stained with haematoxylin and eosin (H&E) or wheat germ agglutinin (WGA) (Deb et al., 2023, 2024). Picrosirius red staining was performed to quantify fibrosis. Apoptosis was assessed using terminal deoxynucleotidyl transferase‐mediated dUTP nick end labelling (TUNEL). H&E and picrosirius red slides were scanned using a PrimeHisto XE histology slide scanner (Carolina Biological, Burlington, NC, USA). WGA slides were imaged using an Agilent BioTek Cytation 1 Cell Imaging Multi‐Mode Reader (Agilent Technologies, Santa Clara, CA, USA). TUNEL slides were imaged using a Leica Stellaris 5 confocal microscope with a 10X objective (Leica Microsystems, Deerfield, IL, USA).

### 2.5 Isolation of cardiomyocytes

Mouse hearts were excised under deep anaesthesia for isolation of ventricular myocytes by enzymatic digestion using Liberase TH (Roche, Basel, Switzerland). Excised hearts were cannulated via the aorta and mounted on a Langendorff perfusion system, then perfused with Tyrode solution at 37°C containing 140 mM NaCl, 10 mM HEPES, 5.6 mM glucose, 5.4 mM KCl, and 0.5 mM MgCl_2_ (pH 7.3 adjusted with NaOH) for 3 min. Hearts were subsequently digested by perfusion with Tyrode solution containing Liberase TH for 10–15 min. After digestion, ventricular myocytes were isolated and plated onto laminin‐coated coverslips for subsequent imaging experiments.

### 2.6 Fluorescence imaging

Live‐cell imaging experiments were performed using an inverted epifluorescence microscope with a ×40 objective (Olympus IX83, Breinigsville, PA, USA). Images were captured using a Hamamatsu ORCA‐Flash4.0 V3 digital CMOS camera (maximum speed: 100 frames/s) (Hamamatsu Photonics, Hamamatsu City, Japan). Intracellular Ca^2+^ imaging was performed using the Fluo‐3 AM indicator, and mitochondrial reactive oxygen species (ROS) were measured using the MitoSOX Red indicator, as previously described (Tow et al., 2022).

### 2.7 ECG recording

Surface electrocardiography (ECG) was recorded using a PowerLab 4/35 system (ADInstruments, Colorado Springs, CO, USA) in mice under light anaesthesia. Isoflurane was maintained at the lowest concentration necessary to achieve sedation while preserving a stable ECG signal (typically 1–1.3%). Baseline ECG recordings were obtained for 2 min, after which mice received intraperitoneal injections of epinephrine (2 mg/kg) and caffeine (120 mg/kg) to induce arrhythmias. ECG recordings continued for an additional 18 min following drug administration. ECG traces were analysed using LabChart 8 Pro software (ADInstruments). The arrhythmia score was calculated as the ratio of ventricular tachycardia (VT) duration to the total recording time.

### 2.8 Mitochondrial energetics analysis

Mitochondrial respiratory function was assessed using high‐resolution respirometry as previously described, with minor modifications (Deb et al., 2026; Nollet et al., 2023; Shi et al., 2023, 2024; Wust et al., 2015). Briefly, 1–3 mg of left ventricular (LV) myocardium was permeabilized in ice‐cold BIOPS solution (Oroboros Instruments, Innsbruck, Austria) containing 50 µg/mL saponin for 30 min. Samples were then washed twice for 10 min in ice‐cold MiR05 mitochondrial respiration medium (Oroboros Instruments), blotted dry, weighed, and analysed at 37°C using a high‐resolution respirometer (Oroboros Oxygraph‐2k). Respiratory substrates included octanoylcarnitine (0.5 mM) and malate (0.1 mM), followed by sequential addition of ADP (5 mM), glutamate (10 mM), and succinate (10 mM). Maximal respiratory capacity was determined by stepwise titration of carbonyl cyanide *p*‐trifluoromethoxyphenylhydrazone (FCCP; 0.1 mM, 1 µL increments). Complex I was subsequently inhibited with rotenone (0.5 µM), and antimycin A (2.5 µM) was added to inhibit complex III, allowing determination of residual oxygen consumption (non‐mitochondrial respiration), which was subtracted from all measurements. Oxygen flux was normalized to tissue wet weight and expressed as pmol/s/mg.

### 2.9 Statistical analysis

Data normality was assessed using the Shapiro–Wilk test. Data meeting the assumptions of normality and homogeneity of variance were analysed using two‐way analysis of variance (ANOVA), followed by Tukey's multiple comparisons test. Data are presented as means ± standard deviation (SD) unless otherwise indicated. A *P*‐value <0.05 was considered statistically significant.

## 3 RESULTS

### 3.1 Metabolic stress combined with RyR2 leak exacerbates myocyte Ca^2+^ dysregulation

The CPVT model of CASQ2 knockout (CASQ2^−/−^) is a classic genetic arrhythmic model characterized by RyR2 leak (Knollmann et al., 2006). To investigate how metabolic stress affects Ca^2+^ homeostasis in the setting of CPVT, we crossbred the CASQ2^−/−^ model with the genetic type 1 diabetes model, the Akita mouse.

To assess intracellular Ca^2+^ dynamics, we performed fluorescence microscopy using the Ca^2+^‐sensitive indicator Fluo‐3 in isolated ventricular myocytes. Systolic Ca^2+^ transients were measured under baseline conditions (Tyrode solution) and following β‐adrenergic stimulation with isoproterenol (ISO).

Under baseline conditions, CASQ2^−/−^, Akita, and Akita‐CASQ2^−/−^ myocytes exhibited Ca^2+^ transient amplitudes similar to those of wild‐type (WT) controls (Figure 1a,b). While CASQ2^−/−^, Akita, and WT myocytes displayed comparable Ca^2+^ transient decay kinetics, Akita‐CASQ2^−/−^ myocytes showed significantly slower decay kinetics than the other three groups (Figure 1a,c).

**FIGURE 1:**
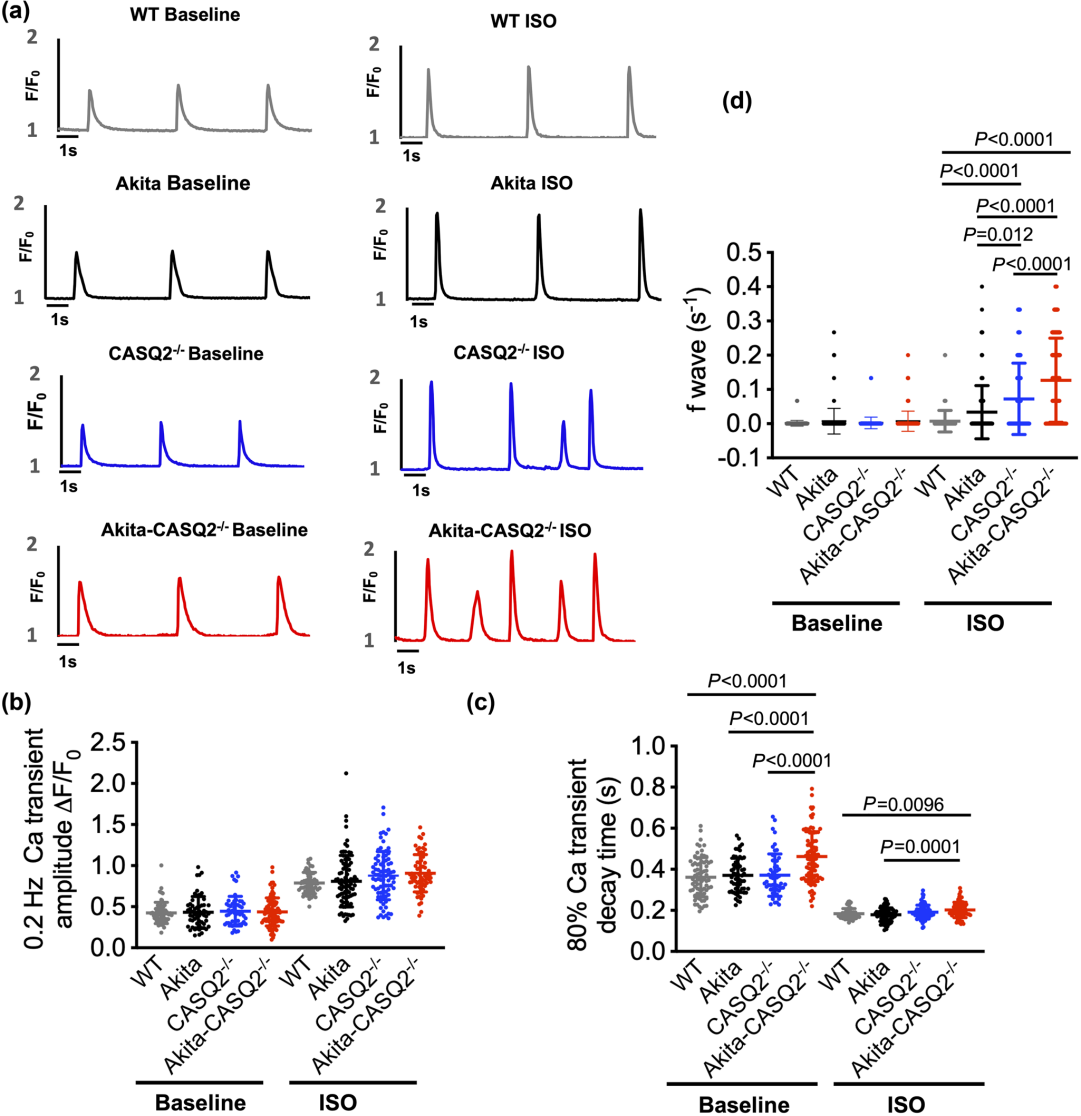
Dysregulation of myocyte Ca^2+^ handling was exacerbated in Akita‐CASQ2^−/−^. (a) Representative traces of time‐dependent fluorescence profiles of Ca^2+^ transient under baseline conditions and in the presence of 100 nM ISO. (b, c) Mean ± SD of average Fluo‐3 amplitude (Δ*F*/*F*_0_) (*n* = 52–91 cells from *N* = 4–5 mice) and 80% Ca^2+^ transient decay time (*n* = 52–91 cells from *N* = 4–5 mice). (d) Mean ± SD of frequency of Ca^2+^ waves (*n* = 99–133 cells from *N* = 4–5 mice). Data were analysed using two‐way ANOVA followed by Tukey's *post hoc* test.

Following ISO treatment, Ca^2+^ transient amplitude increased significantly in all four groups, with no differences in amplitude among them. The kinetics of Ca^2+^ transient decay were also accelerated in all groups. However, Akita‐CASQ2^−/−^ myocytes continued to exhibit the slowest decay kinetics, which remained significantly different from the WT and Akita (Figure 1a,c).

We next quantified the frequency of diastolic Ca^2+^ waves (DCWs), an important indicator of cellular arrhythmic burden and RyR2 leak (Ho et al., 2014; Hwang et al., 2014; Knollmann et al., 2006; Uchinoumi et al., 2010). Ca^2+^ waves typically develop when SR Ca^2+^ load is increased following β‐adrenergic stimulation. Indeed, in all four groups, under baseline conditions, DCWs rarely occurred (Figure 1a,d). Following ISO treatment, the frequency of DCWs significantly increased in CASQ2^−/−^, Akita, and Akita‐CASQ2^−/−^ myocytes. Consistent with previous studies, CASQ2^−/−^ myocytes displayed a significantly higher frequency of Ca^2+^ waves than WT controls (Knollmann et al., 2006; Tow et al., 2022). The Akita model showed only a slight, non‐significant increase in DCWs compared with WT. In contrast, Akita‐CASQ2^−/−^ myocytes exhibited the highest frequency of DCWs, significantly exceeding that observed in CASQ2^−/−^ myocytes (Figure 1a,d).

Together, these results demonstrate that metabolic stress exacerbates dysregulation of myocyte Ca^2+^ homeostasis in Akita‐CASQ2^−/−^ myocytes by slowing Ca^2+^ transient decay kinetics and increasing diastolic Ca^2+^ release, indicative of enhanced RyR2 leak.

### 3.2 Metabolic stress combined with RyR2 leak exacerbates ventricular arrhythmias

We next performed surface ECG in anaesthetized mice to assess susceptibility to stress‐induced arrhythmias. After baseline recordings without pharmacological stimulation, mice were injected with epinephrine and caffeine to provoke arrhythmias.

WT mice exhibited occasional ectopic beats and bigeminy but did not develop more severe ventricular arrhythmias such as VT (Figure 2a,b). In contrast, CASQ2^−/−^ mice displayed sustained episodes of VT, consistent with the characteristic CPVT phenotype observed in vivo (Knollmann et al., 2006; Liu et al., 2015).

**FIGURE 2:**
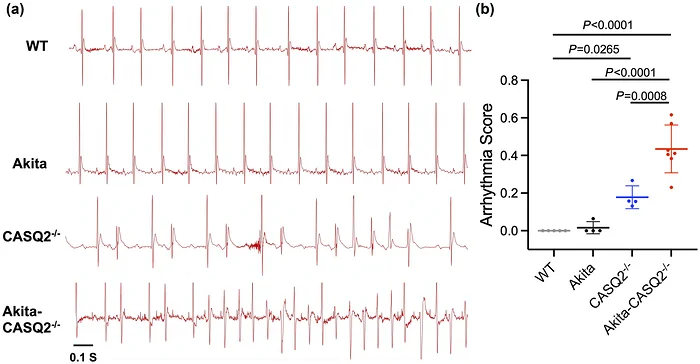
Akita‐CASQ2^−/−^ mice exhibited the most severe arrhythmic burden. (a) Representative surface ECG traces of WT, Akita, CASQ2^−/−^, and Akita‐CASQ2^−/−^ after catecholamine challenge (2 mg/kg epinephrine and 120 mg/kg caffeine, i.p. injection). (b) Mean ± SD of arrhythmia score, *N* = 4–7 mice. Data were analysed using two‐way ANOVA followed by Tukey's *post hoc* test.

Among Akita mice, only one animal exhibited episodes of VT, whereas the remaining mice showed nearly normal ECG recordings. Overall, the Akita group exhibited minimal arrhythmic activity (Figure 2a,b), consistent with the limited increase in DCW frequency observed in the Ca^2+^ imaging experiments (Figure 1d). Previous study also showed that only under programmed ventricular stimulation, VT were more frequently induced in Akita mice than WT (Rajab et al., 2013).

In contrast, Akita‐CASQ2^−/−^ mice displayed severe arrhythmias, characterized by prolonged episodes of VT (Figure 2a,b). Quantitative analysis revealed that Akita‐CASQ2^−/−^ mice had the highest arrhythmia scores, which were significantly greater than those of WT, Akita, and CASQ2^−/−^ mice when analysed by ANOVA. These findings are consistent with the cellular Ca^2+^ imaging results (Figure 1d) and indicate that Akita‐CASQ2^−/−^ mice exhibit the most severe arrhythmias among the four groups.

### 3.3 Metabolic stress combined with RyR2 leak induces hypertrophy without significant cardiac remodelling

To evaluate in vivo cardiac function, we performed echocardiography. Fractional shortening was measured to assess systolic function. All three control groups (WT, CASQ2^−/−^, and Akita) exhibited similar fractional shortening. However, Akita‐CASQ2^−/−^ mice had significantly lower fractional shortening than WT group, indicating mild systolic dysfunction (Figure 3a,b).

**FIGURE 3:**
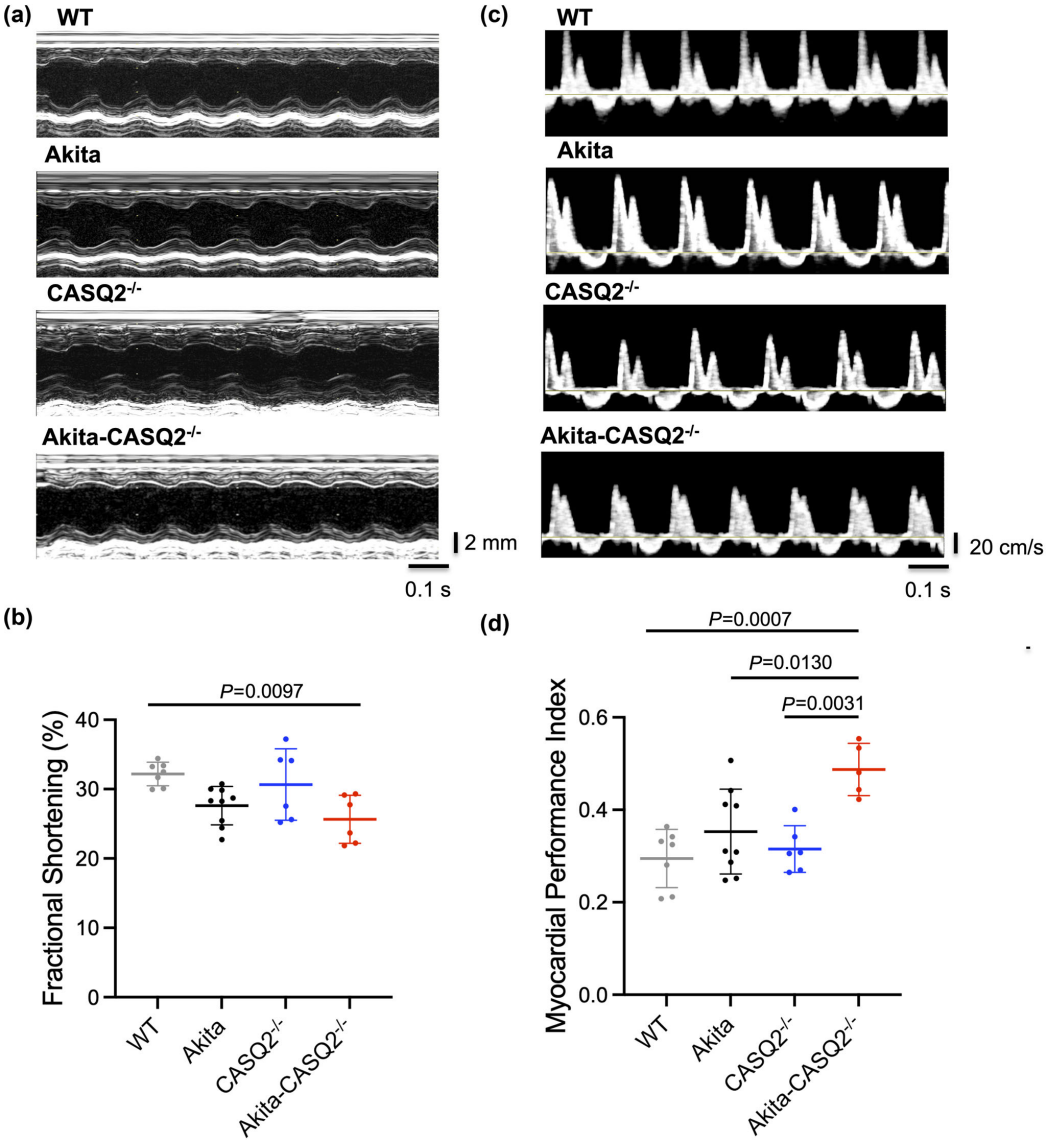
Akita‐CASQ2^−/−^ mice exhibited systolic and diastolic dysfunction. (a) Representative images of M‐mode echocardiography of left ventricle in the different groups. (b) Mean ± SD of fractional shortening (WT, *N* = 7 mice; Akita, *N* = 9 mice; CASQ2^−/−^, *N* = 6 mice; and Akita‐CASQ2^−/−^, *N* = 6 mice). (c) Representative images of Pulse‐wave Doppler. (d) Mean ± SD of myocardial performance index (WT, *N* = 7 mice; Akita, *N* = 9 mice; CASQ2^−/−^, *N* = 6 mice; and Akita‐CASQ2^−/−^, *N* = 5 mice). Data were analysed using two‐way ANOVA followed by Tukey's *post hoc* test.

We next evaluated the MPI, which reflects combined systolic and diastolic function. Higher MPI values indicate impaired LV performance. CASQ2^−/−^ and Akita mice exhibited MPI values comparable to those of WT controls. In contrast, Akita‐CASQ2^−^^/^^−^ mice showed the highest MPI values, which were significantly greater than those of WT, Akita, and CASQ2^−/−^ mice (Figure 3c,d). These findings suggest the presence of diastolic dysfunction in Akita‐CASQ2^−/−^ mice, consistent with the slowed Ca^2+^ transient decay kinetics observed in Akita‐CASQ2^−/−^ myocytes (Figure 1a,c).

Histological analyses were performed to further assess cardiac remodelling. H&E staining revealed no obvious structural abnormalities in Akita‐CASQ2^−/−^ hearts, which exhibited morphology similar to that of WT, CASQ2^−/−^, and Akita hearts (Figure 4a).

**FIGURE 4:**
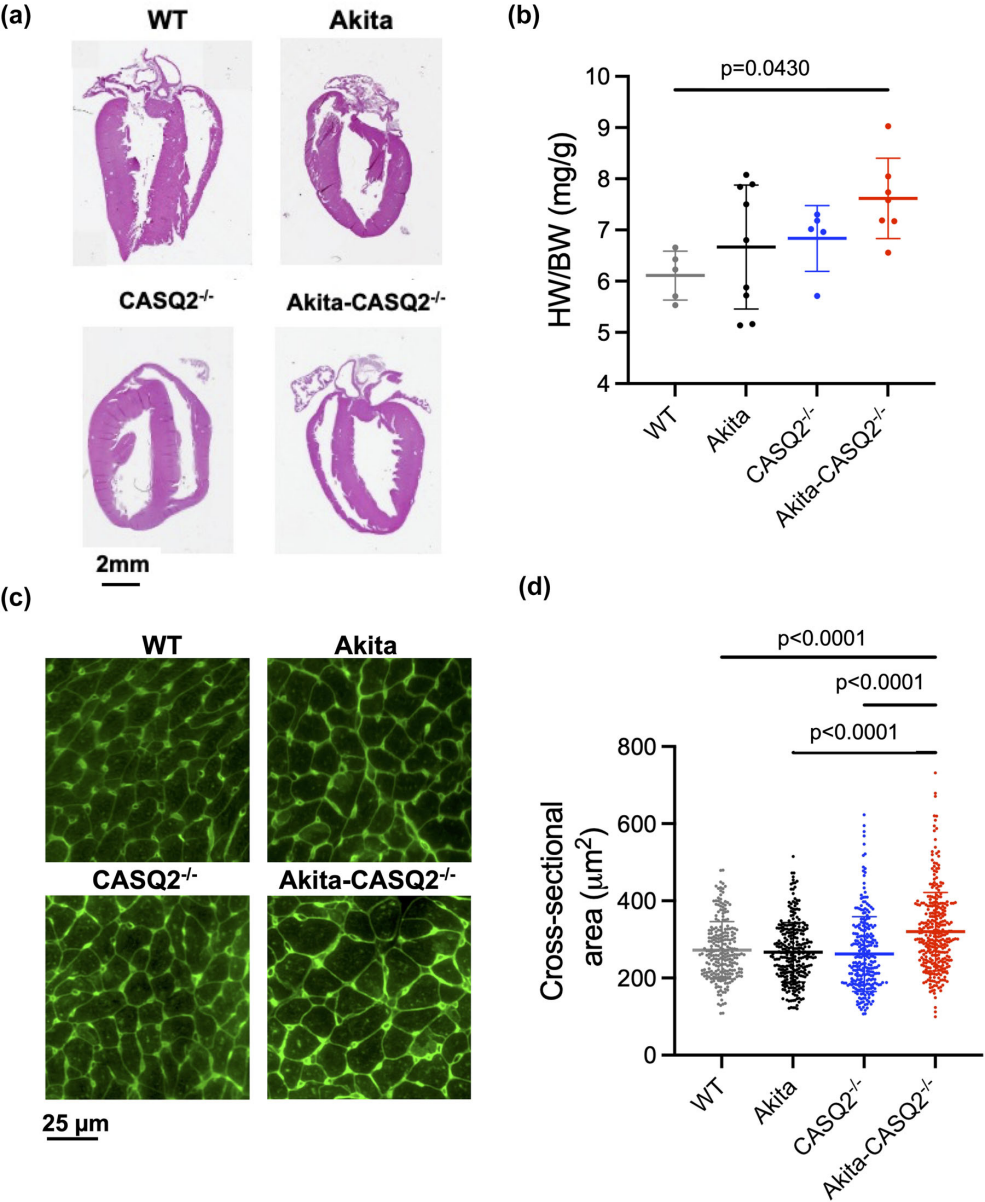
Akita‐CASQ2^−/−^ mice developed cardiac hypertrophy. (a) Representative images of H&E staining of the hearts from WT, Akita, CASQ2^−/−^, and Akita‐CASQ2^−/−^. (b) Mean ± SD of heart weight normalized to body weight (WT, *N* = 5 mice; Akita, *N* = 9 mice; CASQ2^−/−^, *N* = 5 mice; and Akita‐CASQ2^−/−^, *N* = 7 mice). (c) Representative images of the WGA staining. (d) Mean ± SD of cross‐sectional area of myocytes as measured with WGA staining (*n* = 245–317 cells from *N* = 4–5 animals). Data were analysed using two‐way ANOVA followed by Tukey's *post hoc* test.

However, Akita‐CASQ2^−/−^ mice displayed the highest heart weight‐to‐body weight (HW/BW) ratio among the four groups, which was significantly higher than that of WT mice (Figure 4b), suggesting cardiac hypertrophy at the organ level. Consistent with this finding, cardiomyocyte hypertrophy was observed at the cellular level in Akita‐CASQ2^−/−^ hearts, as indicated by an increased myocyte cross‐sectional area measured by WGA staining (Figure 4c,d).

To assess fibrosis, we performed picrosirius red staining. A very low basal level of fibrosis was detected in each group, but no significant differences in fibrosis were observed among WT, CASQ2^−/−^, Akita, and Akita‐CASQ2^−/−^ hearts (Figure 5a,b), suggesting that the basal level of fibrosis detected in Akita‐CASQ2^−/−^ hearts was not indicative of pathological fibrosis.

**FIGURE 5:**
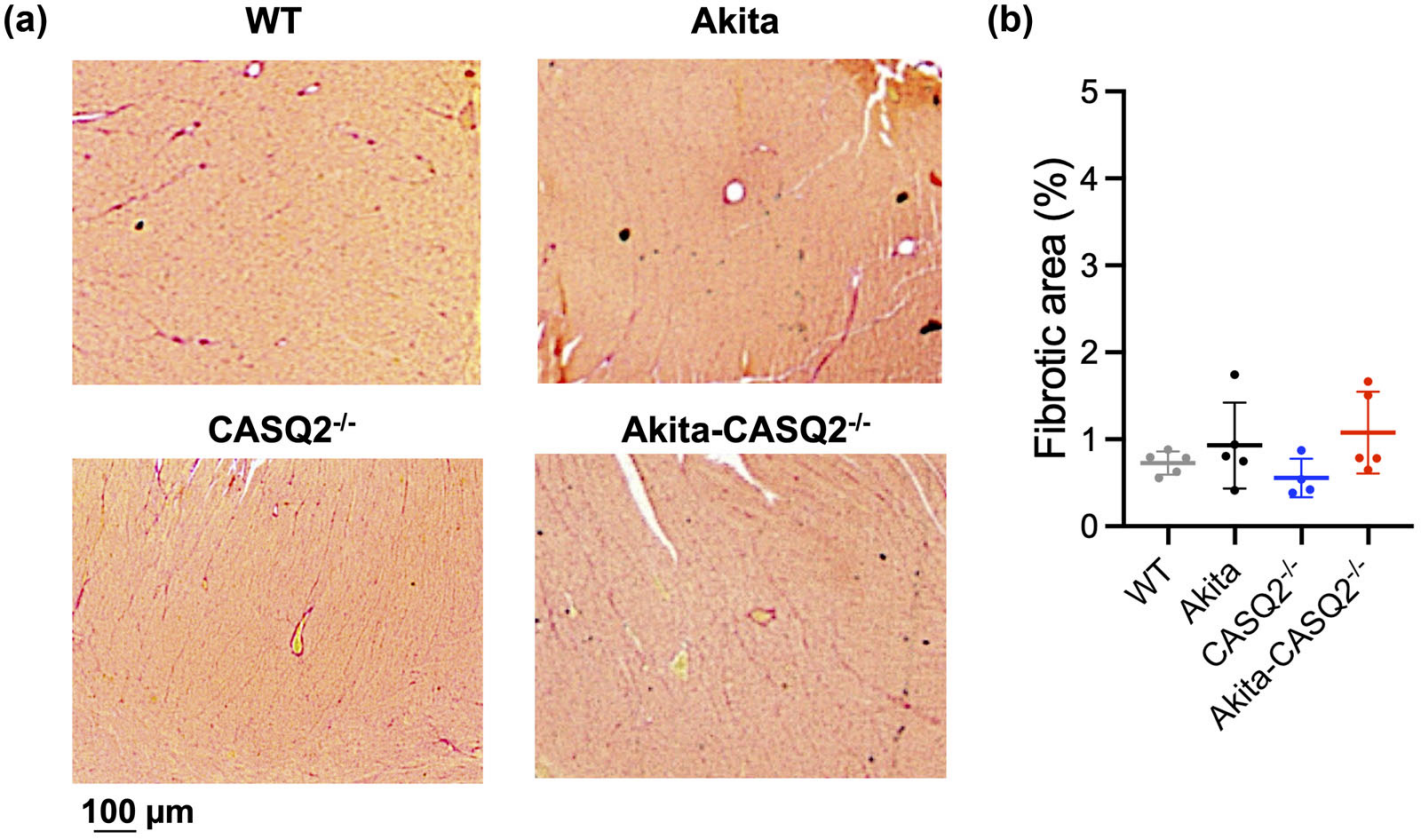
Akita‐CASQ2^−/−^ mice showed limited cardiac fibrosis. (a) Representative images of picrosirius red staining in WT, Akita, CASQ2^−/−^, and Akita‐CASQ2^−/−^. (b) Mean ± SD of quantification of fibrosis normalized to area; *N* = 4–5 mice. Data were analysed using two‐way ANOVA followed by Tukey's *post hoc* test.

Finally, TUNEL assays were performed to evaluate cardiomyocyte apoptosis in fixed cardiac tissue. The validity of the assay was confirmed using a positive control, which produced numerous TUNEL‐positive signals in DNase‐treated tissue (Figure 6a). In contrast, only a limited number of TUNEL‐positive cells were detected in the four experimental groups, with no significant differences among them.

**FIGURE 6:**
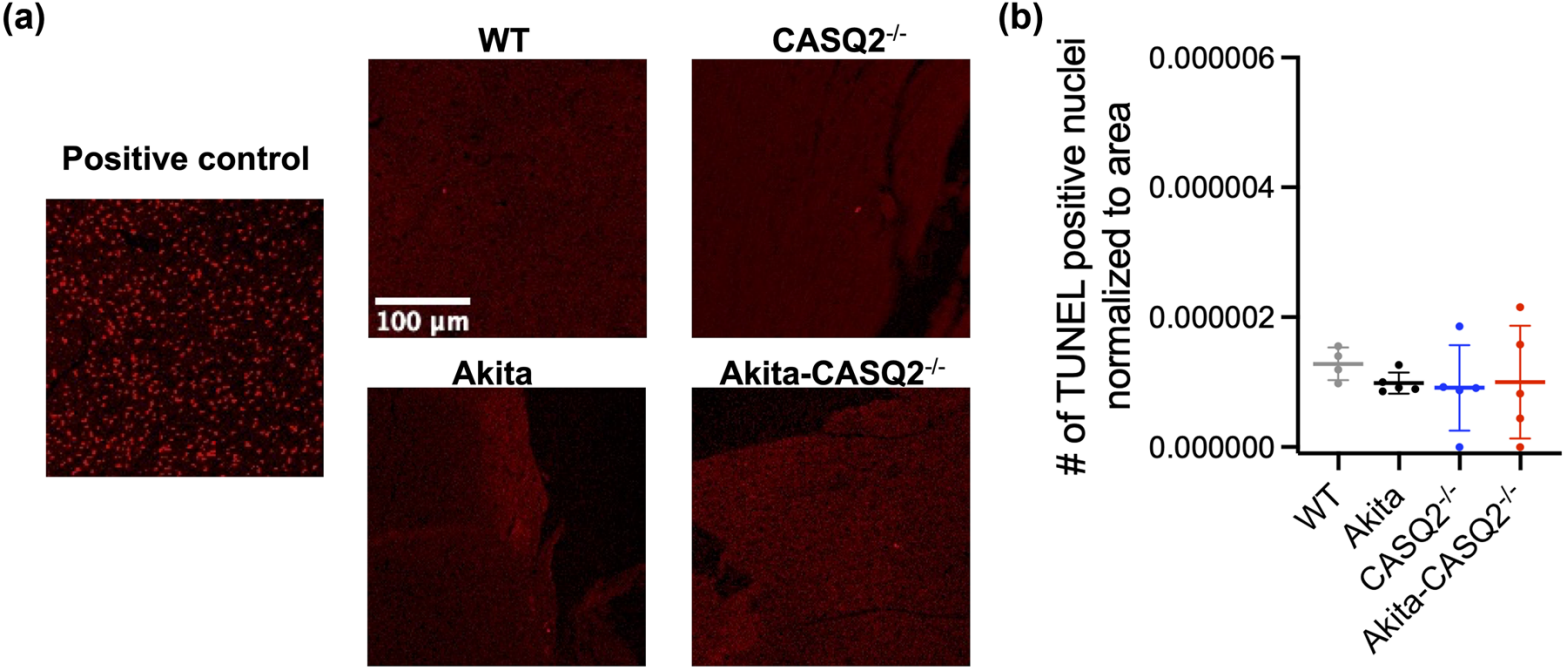
Akita‐CASQ2^−/−^ mice showed few signs of apoptosis. (a) representative images of TUNEL staining in WT, Akita, CASQ2^−/−^, and Akita‐CASQ2^−/−^, and a positive control which is WT tissue treated by DNase. (b) Mean ± SD of quantification of TUNEL positive signal normalized to area; *N* = 4–5 mice. Data were analysed using two‐way ANOVA followed by Tukey's *post hoc* test.

Together, these findings indicate that Akita‐CASQ2^−/−^ hearts exhibit contractile dysfunction and hypertrophy without significant pathological cardiac remodelling, such as fibrosis or cell death.

### 3.4 Metabolic stress combined with RyR2 leak has limited effects on mitochondrial metabolic function

Mitochondrial oxidative phosphorylation (OxPhos) generates approximately 95% of myocardial ATP required for cardiac contraction, with fatty acid oxidation (FAO) supplying up to 60% of this energy demand (Lopaschuk et al., 2021). Compared with WT mice, Akita mice exhibited largely preserved mitochondrial function, with a trend toward increased OxPhos activity mediated by FAO, complex I, and complex II (Figure 7a–c). This preservation of mitochondrial function is consistent with previous observations of enhanced insulin sensitivity in Akita hearts. Although Akita mice display only ∼40% of the circulating insulin levels of non‐diabetic controls, cardiac insulin signalling and inotropic responses remain largely preserved (Bugger et al., 2008).

**FIGURE 7:**
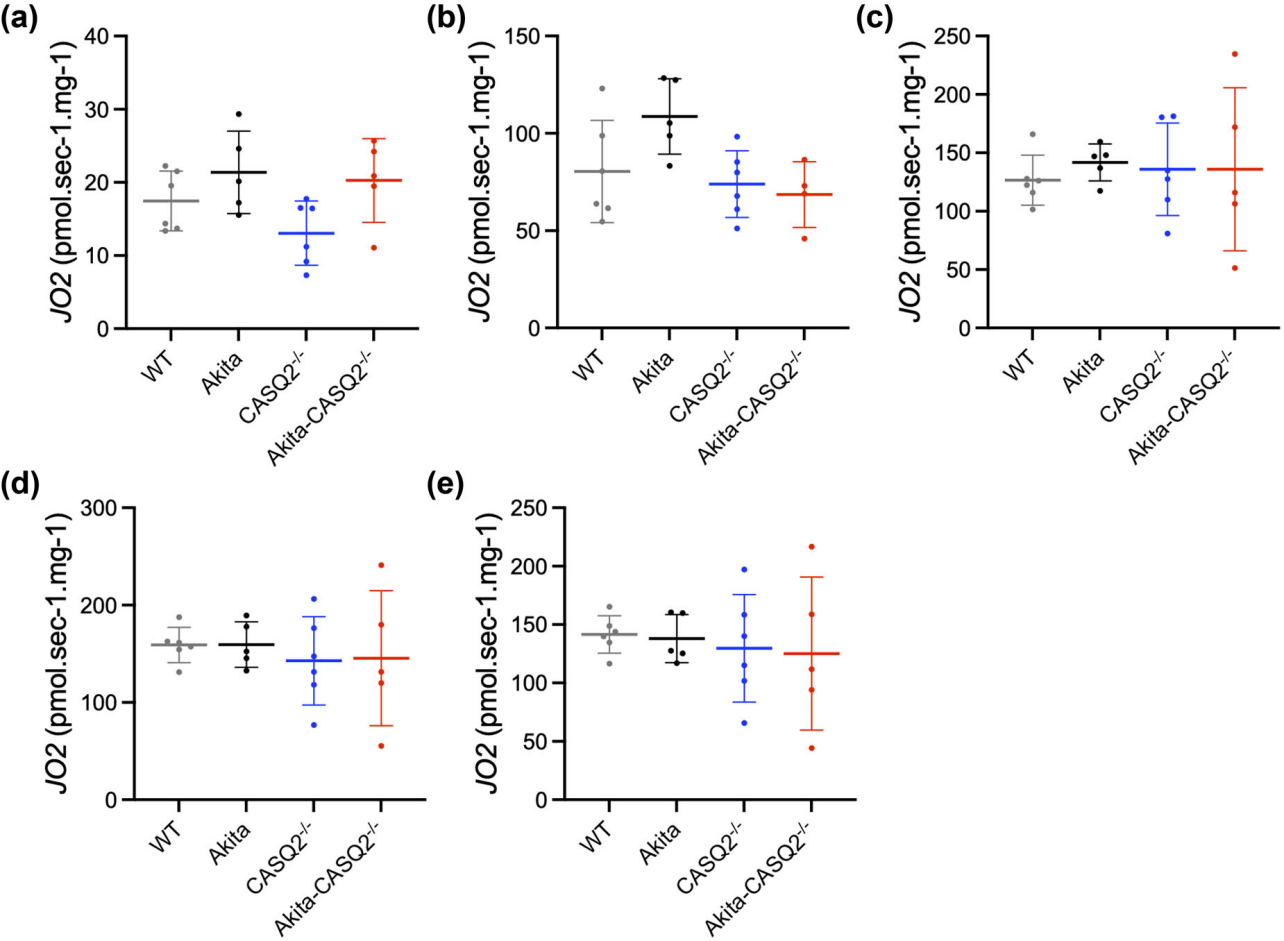
Assessment of mitochondrial function by high‐resolution respirometry (HRR). (a) Basal fatty acid oxidation activity (FAO) of mitochondria in permeabilized myocardium. (b) Fatty acid oxidation activity of mitochondria in permeabilized myocardium in the presence of added ADP (oxidative phosphorylation, OxPhos). (c) The OxPhos activity mediated by FAO, complex I (CI), and complex II (CII). (d) Maximal respiration activity in the presence of FCCP. (e) Spare respiratory capacity of mitochondria in permeabilized myocardium. *N* = 4–6 mice. Data were analysed using two‐way ANOVA followed by Tukey's *post hoc* test.

Measurements of mitochondrial function in CASQ2^−/−^ mice revealed slightly lower OxPhos activity mediated by FAO, complex I, and complex II compared with WT mice; however, these differences were not statistically significant (Figure 7a–c). Additionally, mitochondria from Akita hearts showed a trend toward higher FAO activity (Figure 7a) and ADP‐stimulated FAO (Figure 7b) compared with those from CASQ2^−/−^ hearts. Notably, mitochondria from Akita‐CASQ2^−/−^ hearts did not show significant differences in OxPhos activity compared with those from WT or either of the other two control groups (Figure 7).

### 3.5 Metabolic stress combined with RyR2 leak significantly increases oxidative stress

Elevated oxidative stress is a hallmark of many cardiac pathologies. Therefore, we measured mitochondrial ROS production in cardiomyocytes using the fluorescent indicator MitoSOX Red.

Under baseline conditions, WT and CASQ2^−/−^ myocytes exhibited similar rates of mitochondrial ROS production. In contrast, ROS production was significantly elevated in Akita myocytes. Akita‐CASQ2^−/−^ myocytes displayed an even greater increase in ROS production (Figure 8a,b), with levels exceeding those in all three control groups.

**FIGURE 8:**
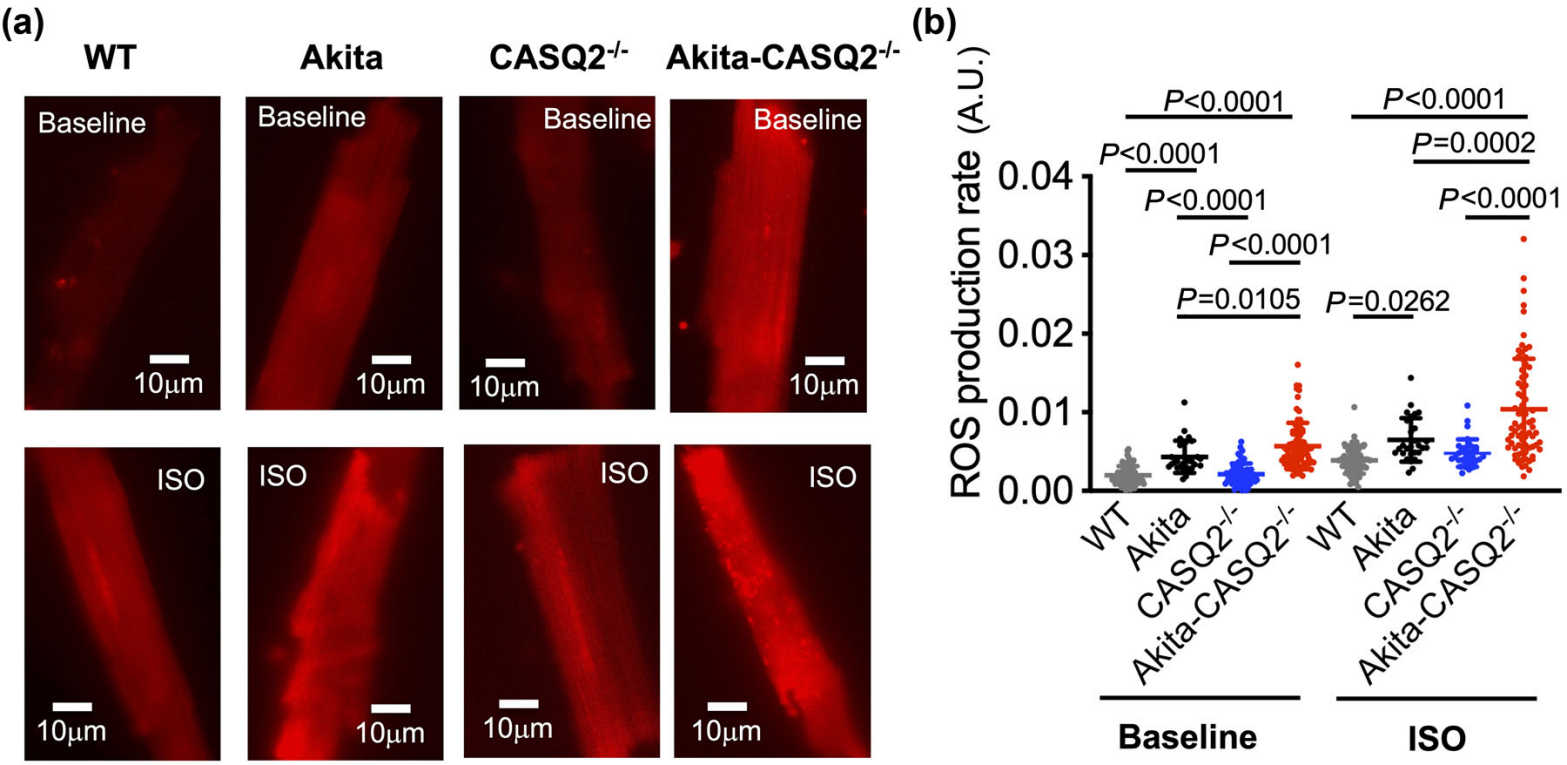
Akita‐CASQ2^−/−^ myocytes had a higher rate of mitochondrial ROS production. (a) representative images of mitochondrial ROS production in WT, Akita, CASQ2^−/−^, and Akita‐CASQ2^−/−^. (b) Mean ± SD of mitochondrial ROS production rate in the four groups; *n* = 29–87 cells from *N =* 3–5 animals. Data were analysed using two‐way ANOVA followed by Tukey's *post hoc* test.

β‐Adrenergic stimulation with ISO significantly increased mitochondrial ROS production in all four groups. Akita myocytes exhibited higher ROS levels than WT controls. Consistent with baseline findings, Akita‐CASQ2^−/−^ myocytes showed the highest ROS production rate, which was significantly greater than that observed in the other three groups (Figure 8a,b).

## 4 DISCUSSION

CPVT is a genetic arrhythmic syndrome characterized by RyR2 dysfunction and diastolic Ca^2+^ leak. In this study, we investigated how metabolic stress influences the pathophenotype of CPVT by crossbreeding a CPVT model (CASQ2^−/−^ mice) with a genetic model of diabetes (Akita mouse). At the cellular level, metabolic stress further exacerbated dysregulation of myocyte Ca^2+^ handling. Akita‐CASQ2^−/−^ myocytes exhibited slower Ca^2+^ transient decay and more severe RyR2 leak, indicated by an increased frequency of DCWs. Consistent with these cellular abnormalities, in vivo echocardiography and ECG recordings revealed cardiac dysfunction. Akita‐CASQ2^−/−^ mice exhibited both systolic and diastolic dysfunction, and severe ventricular arrhythmias. CPVT hearts are anatomically normal without pathological hypertrophy; however, Akita‐CASQ2^−/−^ hearts displayed hypertrophy at both the organ and cellular levels, although they did not exhibit significant fibrosis or cell death. Mitochondrial energetics were preserved in Akita‐CASQ2^−/−^ hearts. Finally, Akita‐CASQ2^−/−^ myocytes showed elevated oxidative stress. Overall, these findings suggest that metabolic stress combined with RyR2 leak exacerbates cardiac dysfunction.

RyR2 dysfunction (i.e., RyR2 leak) is implicated in multiple cardiac pathologies, including heart failure, arrhythmias, and metabolic heart diseases such as prediabetes and diabetes (Belevych et al., 2013; Deb et al., 2026; Tow et al., 2022). RyR2 leak disrupts myocyte Ca^2+^ homeostasis and alters Ca^2+^ transient morphology, thereby contributing to both systolic and diastolic dysfunction (Belevych et al., 2013). In the present study, we did not observe a significant change in systolic Ca^2+^ transient amplitude in Akita‐CASQ2^−/−^ myocytes. However, Akita‐CASQ2^−/−^ myocytes exhibited a slower rate of Ca^2+^ transient decay, which was associated with the diastolic dysfunction detected by echocardiography. In addition to shaping systolic Ca^2+^ transients, RyR2 leak promotes diastolic Ca^2+^ release in the form of arrhythmogenic Ca^2+^ waves, which can directly trigger life‐threatening cardiac arrhythmias (Gyorke, 2009). CPVT models, characterized by severe RyR2 leak, typically exhibit highly malignant arrhythmias (Knollmann et al., 2006). In contrast, metabolic heart diseases, although associated with increased arrhythmic susceptibility, generally display a milder arrhythmic phenotype compared with CPVT models (Deb et al., 2026; Tow et al., 2022). In our previous studies of prediabetes and a type 1 DbCM model, only ectopic beats were observed during in vivo surface ECG recordings (Deb et al., 2026; Tow et al., 2022). Similarly, the Akita mouse exhibited a relatively mild arrhythmic phenotype. In contrast, sustained VT was commonly observed in the CPVT model, as shown in the present study. The extent of RyR2 leak may partially explain these differences. When assessing the frequency of DCWs, we found that the CPVT model (CASQ2^−/−^) exhibited significantly greater RyR2 leak than the Akita model (Figure 1a,d). Notably, the combination of metabolic stress and genetically mediated RyR2 leak further exacerbated RyR2 dysfunction and cellular arrhythmic potential, as Akita‐CASQ2^−/−^ myocytes displayed a higher frequency of DCWs than CASQ2^−/−^ myocytes. Consistently, Akita‐CASQ2^−/−^ mice also exhibited the most severe arrhythmic phenotype in vivo (Figure 2).

Although RyR2 leak has been implicated in multiple cardiac dysfunctions, its contribution to pathological cardiac remodelling remains less clear than its well‐established role in Ca^2+^‐dependent arrhythmias. Indeed, pathological remodelling is typically absent in CPVT models, which generally do not exhibit chamber dilation or hypertrophy, contractile dysfunction, fibrosis, or cell death. Several factors may explain the lack of structural remodelling in CPVT hearts. One possibility is the extent of RyR2 leak. In our previous work, we demonstrated that increasing RyR2 leak by enhancing SERCA activity in the CASQ2^−/−^ model induced a Ca^2+^‐dependent cardiomyopathy characterized by chamber dilation and contractile dysfunction (Kalyanasundaram et al., 2013; Liu et al., 2014). In addition, adaptive molecular mechanisms may protect CPVT hearts from pathological remodelling. Our recent study showed that mitochondria can buffer diastolic Ca^2+^, thereby protecting CASQ2^−/−^ hearts from structural remodelling. Consistent with this idea, ablation of MCU, the primary mediator of mitochondrial Ca^2+^ uptake, in the CASQ2^−/−^ model resulted in premature death and cardiac hypertrophy (Deb et al., 2024; Tow et al., 2022). These findings suggest that RyR2 leak, when combined with additional stressors, may promote pathological remodelling. The Akita mouse, which exhibits hyperglycaemia and metabolic dysfunction, does not by itself develop pronounced cardiac remodelling or overt cardiac dysfunction (Basu et al., 2009). Thus, this model provides an opportunity to test the hypothesis that metabolic stress can promote cardiac remodelling in the setting of RyR2 leak. However, although Akita‐CASQ2^−/−^ hearts developed both systolic and diastolic dysfunction, they exhibited mild cardiac hypertrophy without significant fibrosis or cell death. Nevertheless, Akita‐CASQ2^−/−^ mice displayed more severe overall cardiac dysfunction, which was associated with increased oxidative stress (Figure 8) in this model. Notably, mitochondrial metabolic function remained largely preserved in the Akita‐CASQ2^−/−^ hearts, despite increased mitochondrial ROS production. Increased mitochondrial ROS may represent an early manifestation of mitochondrial stress that precedes measurable alterations in oxygen consumption, ATP production, or respiratory capacity. One potential mechanism underlying the increased ROS production is excessive mitochondrial Ca^2+^ uptake resulting from SR Ca^2+^ leak. Mitochondrial Ca^2+^ uptake can stimulate several TCA cycle dehydrogenases, thereby increasing the generation of reducing equivalents and electron flux through the electron transport chain. Under conditions of increased electron flux, a greater fraction of electrons may be diverted to oxygen, resulting in increased ROS production without necessarily causing an overt impairment in mitochondrial bioenergetic function. Consistent with this possibility, previous studies have shown that SR‐derived Ca^2+^ leak through RyR2 can promote mitochondrial Ca^2+^ uptake and stimulate mitochondrial ROS production (Hamilton et al., 2020). Thus, the severe RyR2‐mediated Ca^2+^ leak in Akita‐CASQ2^−/−^ hearts may contribute to the increased mitochondrial ROS production through enhanced mitochondrial Ca^2+^ loading and redox stress. Future studies will be needed to directly test this potential mechanism.

### 4.1 Limitations

One limitation of this study is the relatively limited sample size, particularly for the histological analyses and experiments involving isolated cardiomyocytes. Importantly, we did not detect significant differences in fibrosis or cell death between the groups, nor did we observe an apparent trend toward a difference in these histological measures. Although we cannot exclude the possibility of subtle differences that may not have been detected because of the limited sample size, the absence of an observable effect suggests that any such differences, if present, are likely to be modest and may have limited biological relevance. Larger studies will be needed to definitively exclude small effects. For the myocyte experiments, although a relatively large number of cells were analysed, these cells were obtained from a limited number of animals, as indicated in the corresponding figure legends. Importantly, individual cells from the same animal cannot be considered fully independent biological replicates. Therefore, these findings should be interpreted with caution and confirmed in future studies using a larger number of animals. This study included only male mice because male Akita mice develop more pronounced hyperglycaemia (Yoshioka et al., 1997). Therefore, the findings of the current study may not be generalizable to female mice, and future studies including both sexes will be needed to determine whether the observed effects are sex‐dependent.

### 4.2 Conclusions

Our results demonstrate that the combination of RyR2 leak and metabolic stress exacerbates cardiac dysfunction. These findings suggest that conditions associated with metabolic stress, such as hyperglycaemia, can worsen RyR2‐mediated cardiac abnormalities.

## AUTHOR CONTRIBUTIONS

*Conceptualization*: Bin Liu. *Data curation*: Valeria Gomez, Arpita Deb, Zhuoxin Li, Yujia Cheng, Benjamin K. Fischer, Jinying Yang, Jeongmin Lee, and Zhiyong Cheng. *Formal Analysis*: Arpita Deb, Valeria Gomez, Zhuoxin Li, and Bin Liu. *Funding Acquisition*: Bin Liu. *Methodology*: Bin Liu and Zhiyong Cheng. *Resources*: Bjorn C. Knollmann and Bin Liu. *Supervision*: Zhiyong Cheng and Bin Liu. *Writing—original draft*: Bin Liu. *Writing—review and editing*: Zhiyong Cheng, Bjorn C. Knollmann, and Bin Liu. All authors have read and approved the final version of this manuscript and agree to be accountable for all aspects of the work in ensuring that questions related to the accuracy or integrity of any part of the work are appropriately investigated and resolved. All persons designated as authors qualify for authorship, and all those who qualify for authorship are listed.

## CONFLICT OF INTEREST

None declared.

## GENERATIVE AI STATEMENT

No generative artificial intelligence (GAI) tools were used in the study design, data collection, data analysis, interpretation of results, and preparation of the manuscript.

## Data Availability

The data that support the findings of this study are included in the article.
